# The Royal Eye Hospital

**Published:** 1902-03-29

**Authors:** 


### THE ROYAL EYE HOSPITAL.

### AN INVISIBLE ATTENDANCE.

A short time ago we heard of a "microscopic attendance," to-day we have to record an invisible one. On Wednesday afternoon, the 19th inst., after post-cards had been sent to the governors, and a notice inserted in three leading papers, a large empty ward, swept and garnished with carbolic, was prepared for the annual meeting of governors of the Royal Eye Hospital, St. George's Circus, S.E. This hospital has a rule which is attended with some danger, *i.e.*, the following persons have the right to vote by proxy:—Patrons, president, peers (spiritual and temporal), members of the House of Commons, judges of the Supreme Court of Judicature, and ladies and governors residing ten miles or more from the hospital. Apparently the entire body of governors, including the council and all the other officers, depended on ten such proxy votes being sent in, for not a single person put in an appearance on Wednesday, except three representing the Surgical staff, the Administration, and the Press.

The Press was the first to arrive. At a few minutes to four o'clock, the deputy-secretary (the secretary being unwell) followed, armed with official documents. At four o'clock the senior surgeon hurried up with a sheaf of blue signed and stamped proxy papers.

"Will you have a quorum?" the Press inquired, somewhat satirically.

"Quorum? oh dear, yes," said the senior surgeon, heartily, throwing the blue papers on the table. "Quite a large attendance—thirty-one."

Having voted himself into the chair, he called upon the deputy secretary to read the minutes of the last meeting, after which he proceeded: "Is it your wish that I sign the minutes? To the contrary? Carried unanimously."

The report was taken as read, and the chairman made some rapid comments for the benefit of the Press, and particularly called attention to the fact that not a penny of the income was spent on advertising the charity. "A tree," he said, "is known by its fruit." The Press industriously took notes, but there was nothing worthy of record beyond what the report stated, and finally the chairman said: "I think it is agreed that the annual report, together with the audited accounts, be adopted and printed for circulation among the governors. All in favour, please signify? To the contrary? Carried."

The Chairman then re-elected the officers for the year, passed the usual votes of thanks, and the meeting adjourned.

"There is usually a vote of thanks to the chair," the deputy secretary suggested, gently.

"No, sir," said the chairman. "I can do many things, but not that." "It is not often," he added, comfortably, addressing the Press, "that you get such a unanimous meeting as this."

### THE YEAR'S WORK.

The death of Sir Francis de Winton, treasurer of this hospital since 1893, is a great loss, and the report for 1891 says that it was largely owing to his inflexible principle that debt was no more justifiable in a charity than in an individual, that the financial position of the hospital improved steadily under his critical watchfulness. "Without incurring debt, by strict economy, with every regard to efficiency, all the beds are now open, and the year closed with a small balance of £26 16s. 11d. in hand." This balance, however, would not have been possible but that a legacy of £173 10s. was reckoned as income rather than capital. The Festival Committee realised £1,745; this included a special donation of 700 guineas "in memoriam the incomparable Queen woman," which has been invested in Sheffield Corporation stock. The total receipts were £3,453 16s. 9d., and the total expenditure £3,426 19s. 10d. Patients' payments amounted to £577 11s. 4d., £436 9s. of this being from in-patients. The average cost of each patient (there were 18,045 during the year) was 3s. 7d.; that is, 625 persons, including those in hospital at the beginning of the year, were treated as in-patients, the average cost being £2 12s. Thus £1,650 was the total sum spent on them. This leaves £1,777 as the cost of 17,440 new out-patients, giving an average of less than 2s. 1d., "the minimum amount," says the report, "that most readers of this spend every month upon newspapers alone." The analysis shows an increase per head upon in-patients, and a decrease on out-patients. This increase is traceable to a longer average stay in hospital; in 1900 it was 14·8, last year 19·5; the reason given being that the strain of poverty upon the poor of South London, owing to the absence of so many bread-winners in South Africa, has been greater than usual, and a longer treatment has consequently been necessary.

Four single bedrooms, which may, with future extensions, be required for additions to the staff, have been again occupied by paying patients, introduced by the hon. surgical officers. This has resulted in increased interest and knowledge of the hospital's good work, and has brought new friends and supporters.

The temperance restaurant premises on the entrance floor continue to be managed by the Vicar of St. Paul's Church, and are a great boon to out-patents and their friends. The friction between the hospital and the London School Board, which "swamped the out-patients' department with thousands of children per week in the Christmas holidays, and endeavoured to extort free medical certificates from hospital doctors," has been removed, as the Board has recently appointed six or seven medical practitioners to supervise the children's eyesight, and to advise as to their need of glasses and fitness to attend school. The out-patients' department continues to be kept open both morning and afternoon, to the great advantage of the poor.

Coronation seats are offered at prices ranging from one guinea (standing room) to thirty guineas and upwards, and a good many are already booked. All proceeds go to the hospital.

The report closes with a reference to the population of London south of the Thames, and the relative number of free beds. The population is stated at 1,664,004, and the free hospital beds at 986, as compared with a population of 2,768,914 north of the river, with 3,975 free beds, a proportional deficiency of fully 1,400 free beds for the very poor sufferers in South London.

